# A Digital Microfluidics Platform for Loop-Mediated Isothermal Amplification Detection

**DOI:** 10.3390/s17112616

**Published:** 2017-11-16

**Authors:** Beatriz Jorge Coelho, Bruno Veigas, Hugo Águas, Elvira Fortunato, Rodrigo Martins, Pedro Viana Baptista, Rui Igreja

**Affiliations:** 1 CENIMAT|i3N, Departamento de Ciência dos Materiais, Faculdade de Ciências e Tecnologia, Universidade NOVA de Lisboa, Campus de Caparica, 2829-516 Caparica, Portugal; bj.coelho@campus.fct.unl.pt (B.J.C.); bmrveigas@gmail.com (B.V.); hma@fct.unl.pt (H.A.); emf@fct.unl.pt (E.F.); rfpm@fct.unl.pt (R.M.); 2 UCIBIO, Departamento de Ciências da Vida, Faculdade de Ciências e Tecnologia, Universidade NOVA de Lisboa, Campus de Caparica, 2829-516 Caparica, Portugal

**Keywords:** digital microfluidics, loop-mediated isothermal amplification, *c-Myc*, lab-on-a-chip, point-of-care diagnostics

## Abstract

Digital microfluidics (DMF) arises as the next step in the fast-evolving field of operation platforms for molecular diagnostics. Moreover, isothermal schemes, such as loop-mediated isothermal amplification (LAMP), allow for further simplification of amplification protocols. Integrating DMF with LAMP will be at the core of a new generation of detection devices for effective molecular diagnostics at point-of-care (POC), providing simple, fast, and automated nucleic acid amplification with exceptional integration capabilities. Here, we demonstrate for the first time the role of coupling DMF and LAMP, in a dedicated device that allows straightforward mixing of LAMP reagents and target DNA, as well as optimum temperature control (reaction droplets undergo a temperature variation of just 0.3 °C, for 65 °C at the bottom plate). This device is produced using low-temperature and low-cost production processes, adaptable to disposable and flexible substrates. DMF-LAMP is performed with enhanced sensitivity without compromising reaction efficacy or losing reliability and efficiency, by LAMP-amplifying 0.5 ng/µL of target DNA in just 45 min. Moreover, on-chip LAMP was performed in 1.5 µL, a considerably lower volume than standard bench-top reactions.

## 1. Introduction

Point-of-care (POC) testing relies greatly on microfluidics to shift the paradigm of molecular diagnostics, from the centralized structure of highly specialized laboratories, to perform diagnostics close to the patient. This is particularly relevant in cancer biomarkers, where prompt diagnostics is crucial for effective therapeutic regimens that require molecular follow-up to evaluate disease progression. Digital microfluidics (DMF) has recently emerged as a powerful tool for point-of-care diagnostics, namely in the field of nucleic acid amplification [1]. DMF relies on an electrode array architecture able to establish electric potentials on electrodes, which allows controlling discrete microliter droplets with high precision in integrated systems, via computed control commands [2,3]. Each droplet can be considered as a micro-reactor leading to an enhancement of the efficiency of chemical and biological reactions that take place [4]. DNA amplification in DMF devices shows several advantages, namely reagent consumption reduction up to 40,000-fold [5], improved detection limit up to 100 times [6] versus bench-top processes, faster reaction time by 50% [7], and the possibility of fully automated processes, thus reducing costs and cross-contamination.

Most DMF platforms for DNA amplification rely on polymerase chain reaction (PCR), which requires thermal cycling (e.g., peltier) and therefore increasing complexity [5,6,7,8,9,10]. More recently, DMF has been integrated with isothermal DNA amplification approaches, such as circle-to-circle amplification [11] or recombinase polymerase amplification [6].

Other robust isothermal amplification strategies have been highlighted by the World Health Organization (WHO) towards diagnostic tests at peripheral health centers focusing on optimal detection with minimal laboratory infrastructure [12]. Loop-mediated isothermal amplification (LAMP) allows a 10^9^-fold amplification of a target DNA sequence under one hour at a relatively low temperature (60 °C to 65 °C) [13]. Due to their intrinsic capabilities, integration of LAMP and DMF should provide several advantages over bench-top reactions in terms of operation, in particular for POC genetic diagnostics [14,15,16]. Moreover, integrating DMF and LAMP could significantly lower the costs associated with molecular diagnostics of cancer, allowing for quantification that assists clinicians in prognosis and/or prediction of clinical outcome.

*c-Myc* (8q24.2.11; OMIM:190080) is one of the most frequently overexpressed oncogenes involved in 20% of all human cancers [17]. Both genomic and functional analyses of *c-Myc* suggest that it behaves as a global regulator of transcription, cell cycle regulation, metabolism, ribosome biogenesis, protein synthesis, and mitochondrial function. It is also involved in the repression of genes involved in cell growth arrest and cell adhesion [17]. Although *c-Myc* overexpression is not related to any specific kind of cancer, recent studies provide the evidence of the prognostic value of *c-Myc* and associated genes, suggesting its use as a cancer biomarker [18,19].

**Figure 1:**
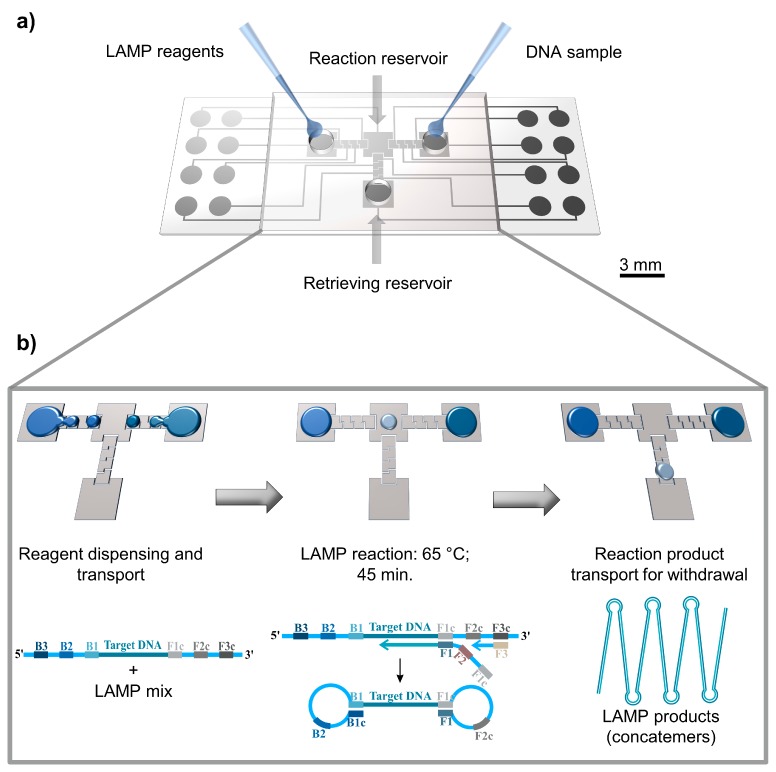
Digital microfluidics with loop-mediated isothermal amplification (DMF-LAMP). (**a**) Schematic representation of a DMF chip, where reagents are inserted by using a simple pipette. (**b**) Main steps required to perform a LAMP reaction on the device. LAMP reagents and DNA sample are inserted in opposite ends of the chip, and aliquots (droplets) of each are then withdrawn from the main reservoirs and conducted to the central reservoir where LAMP reaction occurs. Finally, LAMP products are recovered from the device.

In the present study, we integrate a specially designed DMF platform with LAMP (Figure 1) for amplification of the *c-Myc* oncogene, which is overexpressed in most human cancers, targeting exploitation of future integrated platforms for POC cancer diagnostics. This T-shaped platform contains designated areas for LAMP reagents, DNA samples, and the mixing of both, as well as a recovery region for reaction product withdrawal, connected with each other with zig-zag electrodes to facilitate droplet movement.

## 2. Materials and Methods

### 2.1. DMF Device Design and Fabrication

A T-shaped chip (see Figure 1) was designed with four major regions: (1) a reservoir for LAMP reagents; (2) a reservoir for DNA sample; (3) a reaction reservoir, where LAMP reagents will be mixed with the DNA sample, and where LAMP reactions will occur; (4) a retrieving reservoir, from which reaction products may be withdrawn from the device. Several electrode designs have been reported in DMF [20,21]; however, in this work, zig-zag crenelated electrodes with a gap between neighboring electrodes of 30 µm were used. This electrode configuration places indentations of one electrode inside the area of another, forcing the droplet to occupy some of the area belonging to the next electrode, thus improving droplet motion by reducing the effect of the hydrophobic gap. For full DMF chip electrode array specifications, see Table S1 and Figure S1a.

In DMF, two major configurations are used: (1) open format, where droplets move over a single substrate [22]; and (2) closed format, where droplets move between two substrates [2]. The closed format was chosen for this work, since it allows for a wider range of fluidic operations (dispensing, splitting, merging, mixing). The device developed here is constituted by four elements: a substrate, electrodes, a dielectric layer, and a hydrophobic layer. The bottom-plate electrodes were designed using CorelDraw X7^®^ software (Corel Corporation, OT, Canada) and printed on high-resolution emulsion film photomasks (JD Photodata, Hitchin, UK). These masks contained the pattern for the chromium electrodes, connection lines and pads, which were patterned on glass substrates by photolithography. After cleaning, glass substrates were covered with AZ6632 1.2 μm grade photoresist (MicroChemicals, Ulm, Germany) by spin-coating (Model WS-650MZ-23NPP–Laurell, North Wales, PA, USA) at 2000 rpm for 10 s, followed by 4000 rpm for 20 s. The substrates were then heated (Isotemp–Fisher Scientific, Waltham, MA, USA) at 115 °C for 75 s. Following the prebake process, substrates were exposed (Karl Suss, Garching, Germany) to UV radiation for 5 s. Then, the photoresist was developed using the AZ726 MIF developer (MicroChemicals, Ulm, Germany) for 35 s and substrates immersed in pure water to stop the developing process and dried by a nitrogen jet. After patterning, a 200 nm chromium layer was deposited at 100 °C in a homemade electron-beam (e-beam) evaporation system. Following chromium deposition, lift-off was performed. A 2 μm layer of dielectric, Parylene C (CAS 28804-46-8), was then deposited onto the substrates by chemical vapor deposition (SCS Labcoater^®^-PDS 2010, Indianapolis, IN, USA). Finally, a 50 nm hydrophobic layer of Teflon-AF 1600 (DuPont, Wilmington, DE, USA) was deposited over the dielectric layer. For this, a solution containing 0.6% wt/wt of Teflon^®^ AF 1600 in Fluorinert FC-40 (DuPont, Wilmington, DE, USA) was spin-coated at 1000 rpm for 30 s with a coating acceleration of 100 rpm/s, followed by baking at 160 °C for 10 min.

The top plate of the microfluidic device consisted of a glass substrate coated with a 100 nm layer of indium tin oxide (ITO), in which inlet/outlet ports (drilled with a diamond tip) partially overlapping the reservoir areas, allowing sample or reagent insertion/withdrawal to/from the chip, were mechanically drilled with a diamond tip. This plate was further coated with Teflon^®^, following the procedure described above. The gap between plates was maintained at approximately 180 μm, by using 3 layers of Kapton^®^ tape (PPC216–Farnell, Leeds, UK). Plate separation was measured by a digital micrometer (IP65–Mitutoyo, Kawasaki, Japan). Lastly, the full chip assembly was filled with 5 cSt silicone oil (Sigma–Aldrich, St. Louis, MO, USA) immediately before use. For an overview of the chip assembly, see Figure S1a,b.

### 2.2. DMF Control System

The device was mounted on a home-made 3D printed support with connection pins, based on an open-source DMF control system [23], modified to include a glass piece with a thin film of ITO thermoresistor (as heating element). An HD camera (Microsoft LifeCam Studio) was used to enable real-time visualization of all fluidic operations. Electrodes can be activated/deactivated via a Matlab^®^ script, which transmits the information to an Arduino Mega board connected to a Voltage Switching Unit adapted from the open-source control system [23], which enables an ON state (AC voltage) or OFF state (ground) for each electrode/reservoir. A function generator (TG1000 10 MHz DDS–TTi), connected to a voltage amplifier (PZD700A–TREK) were used to generate the AC voltage signals. The output current from the amplifier was monitored to evaluate the real-time system impedance. For an overview of the control system, see Figure S2.

### 2.3. Temperature Control System

Temperature control relies on a custom-designed thin ITO film resistor (3 × 3 cm^2^) placed under the bottom plate. This resistor is able to provide a maximum heating power of 4.1 W, which is transferred through the bottom plate to the reaction droplet, allowing a process temperature control from room temperature up to 100 °C with a precision of ± 0.5 °C. The resistor is connected to the output of a power driver, which in turn is controlled by a proportional–integral–derivative (PID) controller (made in house with an Arduino UNO). A K-type thermocouple glued to the bottom plate is used as a temperature sensor. The PID controller prevents temperature deviations, since it continuously compares the actual temperature with the set point temperature.

The temperature gradient along the vertical axis of a reaction droplet was also assessed using additional temperature information from a second K-type thermocouple placed on the top plate. In all cases, real time temperatures were recorded on both bottom and top plates.

### 2.4. Benchtop LAMP Amplification of c-Myc

A 229 base pair (bp) fragment of the human *c-Myc* proto-oncogene (Ac. No. NM_002467) associated to cancer development [24] was PCR amplified using primers MYCforward and MYCreverse, and the resulting amplicon used as sample for LAMP amplification (see Table S2). PCR amplification was performed in triplicate on a Bio-Rad MyCycler Thermocycler (Bio-Rad, CA, USA) in 20 µL using 1 µM of the specific primers, 0.4 mM dNTPs (ThermoFisher Scientific, Waltham, MA, USA) with 1 U Taq DNA Polymerase (GE Healthcare Europe, Freiburg, Germany), with the following thermal cycling conditions: an initial 5 min denaturation at 95 °C, followed by 19 amplification cycles of denaturation at 95 °C for 30 s, annealing at 66 °C for 30 s, elongation at 72 °C for 30 s, and a final elongation at 72 °C for 5 min.

LAMP was performed as described by Veigas et al. [13,25]. LAMP of the human *c-Myc* proto-oncogene requires four specific primers: a forward outer primer (FP), a backward outer primer (BP), a forward inner primer (FIP), and a backward inner primer (BIP) (Table S2). LAMP primers for *c-Myc* were designed using Primer Explorer V4 (http://primerex- plorer.jp/elamp4.0.0/). The reaction was carried out in a 20 µL reaction mixture containing 0.8 μM of each inner primer FIP and BIP, 0.2 μM of each outer primer FP and BP, 0.4 mM of dNTP mix (ThermoFisher Scientific, Waltham, MA, USA), 1 M betaine (Sigma–Aldrich, St. Louis, MO, USA), 4 mM MgCl_2_ (Sigma–Aldrich, St. Louis, MO, USA), 1× of the supplied buffer, and 0.5 ng/µL to 0.05 pg/µL initial DNA concentration. LAMP reaction was performed with 8 U of Bst DNA polymerase (large fragment; New England Biolabs Inc., Beverly, MA, USA) and allowed to react for 60 min at 65 °C in a Bio-Rad MyCycler Thermocycler (BioRad, CA, USA). As a control, a sample solution submitted to the same reaction procedure without any template DNA was used.

### 2.5. LAMP Reaction Optimization for On-Chip Integration

Adaptation of standard reaction conditions from bench-top LAMP to DMF-LAMP required optimization of reaction conditions, such as a volume reduction and a faster overall reaction time. One of the major concerns about performing LAMP on a DMF platform is the effect that a significant volume reduction might have on the amplification efficiency. Bench-top LAMP reactions usually resort to 20–50 µL reaction volumes; however, microfluidic devices allow volume reductions [4,16,26] down to the nL scale [6,27,28,29]. The DMF-LAMP platform here described was designed to work with much lower reaction volumes, i.e., smaller than 2 µL. To prevent evaporation, 5 µL of mineral oil was added to the reaction tubes. Different target DNA concentrations (0.5 ng/µL down to 0.05 pg/µL), reaction times (30 min to 90 min), and reaction volumes (20 µL down to 1.25 µL) were evaluated to achieve the optimal conditions for on-chip reaction. Furthermore, the target DNA was resuspended on a 1× enzyme buffer (3.97 mS/cm conductivity) to improve on-chip droplet actuation.

### 2.6. DMF-LAMP Amplification of c-Myc (On-Chip)

The overall on-chip protocol is depicted in Figure 1: the reaction mixture was transferred to the left DMF chip reservoir and the DNA sample to the right reservoir through the drilled inlets. LAMP reaction was performed by adding 10 droplets of LAMP mix reagents (1.40 µL) to 1 droplet of DNA solution (0.14 µL), thus guaranteeing a 0.5 ng/µL DNA concentration in a 1.54 µL total reaction volume on the mixing reservoir (central reservoir). The reaction temperature was kept constant at 65 °C (bottom plate temperature). The amplification efficiency was evaluated for 15 min, 30 min, and 45 min.

## 3. Results and Discussion

### 3.1. DMF Device Characterization and Performance

**Figure 2:**
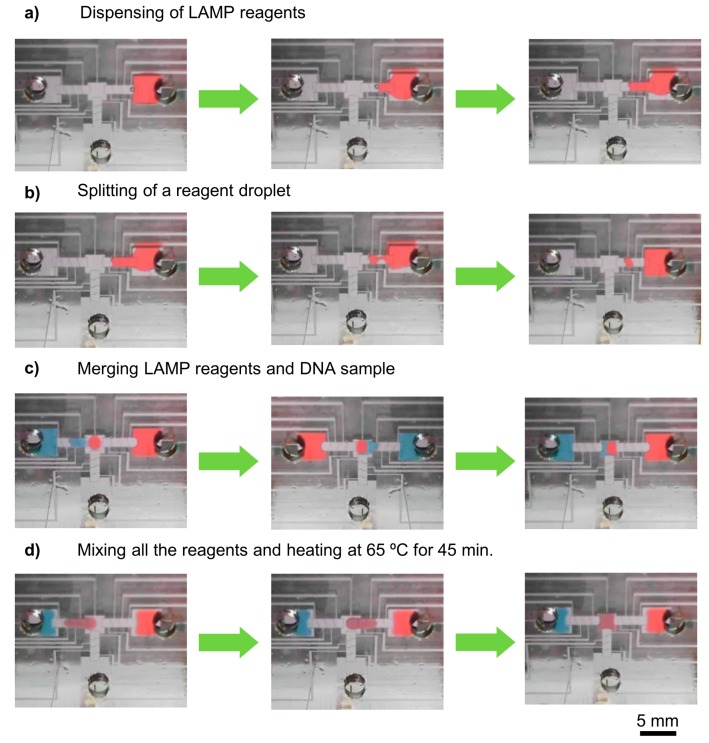
DMF-LAMP procedure. Video frames showing the main steps required for a DMF-LAMP reaction: (**a**) LAMP reagent dispensing from the left reservoir; (**b**) droplet splitting; (**c**) sample merging (LAMP reagents and DNA sample); (**d**) reagent mixing with left/right movements and DMF-LAMP reaction. Reagent droplets were dyed for better visualization. Please note that the mixing reservoir is smaller than usual, as to accommodate only two droplets, for demonstration of a working device.

**Figure 3:**
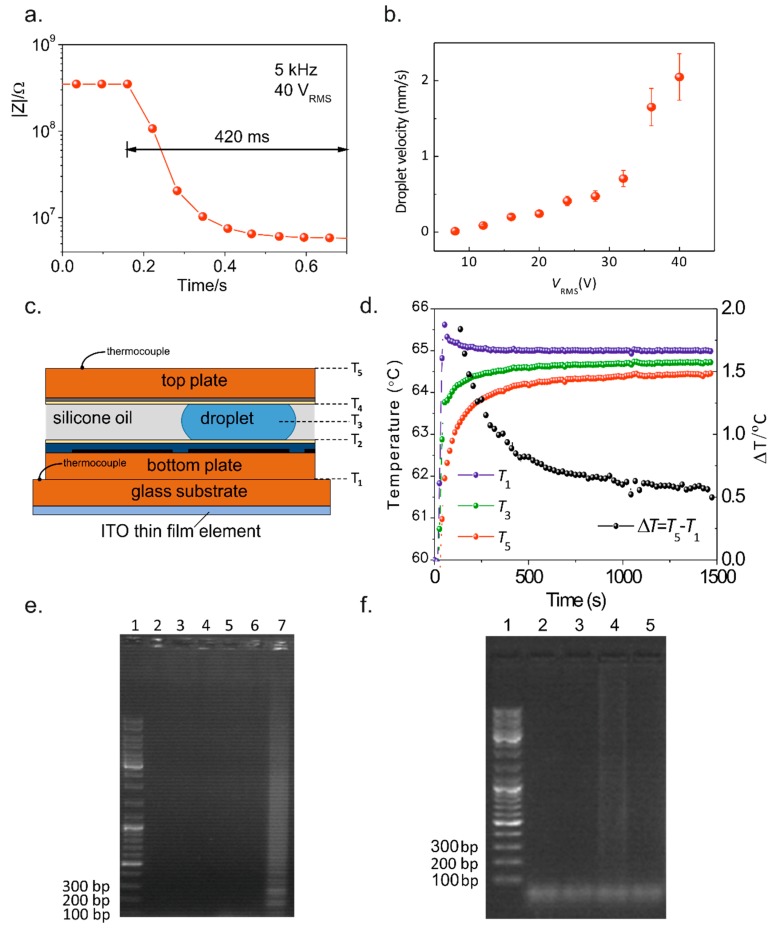
Overall device performance. (**a**) System impedance during the droplet movement from one electrode to the adjacent one. (**b**) Droplet velocity as a function of activation voltage. (**c**) Schematic view of the DMF chip cross-section. (**d**) Transient and steady-state temperature behavior. (**e**) Electrophoretic analysis for on-chip LAMP products (DNA concentration of 500 pg/µL at 65 °C) in comparison with a DNA ladder (Lane 1): 15 min (negative control–Lane 2, positive control–Lane 3), 30 min (negative control–Lane 4, positive control–Lane 5), and 45 min (negative control–Lane 6, positive control–Lane 7). (**f**) Electrophoretic analysis of the bench-top LAMP products (500 pg/µL initial DNA concentration at 65 °C). Lane 1: ladder; Lanes 2 and 4: positive controls for 30 min and 45 min reaction times, respectively; Lanes 3 and 5: negative controls for 30 min and 45 min reaction times, respectively.

The proposed method for sample input/output was proven to allow simple, straightforward reagent insertion and withdrawal to/from the chip, using a pipette (see Figure S3). All fluidic operations (dispensing, splitting, merging, and mixing) were tested and successfully accomplished in this platform for both DNA sample and LAMP reagents, for an actuation voltage of 40 V_RMS_ at 5 kHz (Figure 2), allowing droplet movement at high speed (~2 mm/s), which is compatible with future multiplexing and more complex device architectures. Furthermore, lower voltages (down to 8 V_RMS_) are possible if lower speeds are required (Figure 3b). Droplet velocity was determined by averaging droplet head and tail velocities during the droplet movement from one electrode to the next and by monitoring the system impedance in real-time (Figure 3a). Owing to the extremely fine droplet control attained, the device is able to move low-volume droplets (0.14 µL per droplet), therefore allowing very low-volume LAMP reactions. For more information on the average droplet velocity, see Supplementary Material.

### 3.2. Temperature Control Evaluation on the DMF Platform

To guarantee that LAMP reactions occur within the optimal temperature range (between 60 and 65 °C), the temperature gradient across a reaction droplet was assessed by measuring both bottom and top plate temperatures (see Figure 3c,d and Table S3) and studying their transient and steady-state responses in real time (Figure 3d). When changing the set point, the temperature stabilization in the thin film resistor (T1) proved to be quite fast (a time constant of ~20 s) and the temperature on the top plate (T5) showed a time constant of ~100 s. The PID controller was also found to prevent temperature deviations, since the bottom plate temperature virtually presents no deviations from the set point after reaching the steady-state regime. Moreover, at steady-state, the temperature difference between the bottom- and top-plate is only 0.6 °C for a bottom-plate temperature of 65 °C (Figure 3d).

Additionally, the theoretical evaluation of heat transfer and temperature gradient for a single reaction droplet was performed (see Table S4) aiming to study the temperature gradient within the reaction droplet itself (T2, T3, and T4). With the bottom plate temperature (T1) set to 60 °C during a LAMP reaction, the droplets will undergo a vertical temperature gradient of about 0.5 °C, between T2 = 59.8 °C and T4 = 59.3 °C. However, if the bottom plate temperature (T1) is set to 65 °C, then droplets will undergo a temperature gradient of 0.3 °C, between T2 = 64.9 °C and T4 = 64.6 °C. These results show optimal temperature control for LAMP reactions to occur.

### 3.3. Benchtop LAMP Optimization for On-Chip Integration

LAMP was performed in 20 µL with successive 10-fold dilutions of target DNA, ranging from 0.5 ng/µL down to 0.05 pg/µL with constant temperature (65 °C) and variable reaction times (30 to 90 min). For 60 min reactions, amplification is possible for DNA concentrations >0.05 pg/µL; whereas, for 90 min, the reaction allowed amplification from 0.05 pg/µL (see Figure S4). The effect of the total reaction volume on amplification efficiency was evaluated by a set of reactions at 65 °C, for 60 and 90 min, with an initial DNA concentration of 0.5 ng/µL and volumes ranging from 20 µL down to 1.25 µL (see Figure S5). For both reaction times, DNA amplification was possible until a minimum volume of 2 µL. There were no visible signs of evaporation (air bubbles or reduction of the intended volume), which indicates that using oil to cover the aqueous solution is an effective way to reduce volume loss.

### 3.4. DMF-LAMP

Considering the above-mentioned results, DMF-LAMP conditions were set to 0.5 ng/µL of initial DNA concentration for a reaction time of 60 min, at 65 °C. Furthermore, chip specifications (zig-zag electrode shape, as well as plate spacing) were set to test a low on-chip reaction volume: 1.54 µL (on chip manipulation was possible down to 1.54 µL that still allowed for robust DNA amplification, even though off chip amplification was only feasible down to 2 µL). During DMF-LAMP, the mixing region/area was kept activated to avoid unwanted droplet spreading. This was achieved with lower voltages (20 V_RMS_ at 5 kHz) to prevent any eventual chip damage during LAMP which might affect LAMP efficiency.

Figure 3e shows the electrophoretic analysis for DMF-LAMP products, with different reaction times (15 min, 30 min, and 45 min). There is no observable amplification for 15 min and 30 min, but a distinctive LAMP band pattern was attained for 45 min (Lane 7), indicating successful DNA amplification. Figure 3f shows the electrophoretic analysis for bench-top LAMP products and for different reaction times. The DMF-LAMP amplification products produce a brighter band (Figure 3e, Lane 7) when compared to the same bench-top-based amplification reaction products (normalized band fluorescence intensity of 139.0 a.u. versus 78.8 a.u., respectively) (Figure 3f, Lane 4), which suggests that the on-chip LAMP reaction may be more efficient than the bench-top counterpart. This demonstrates that the DMF platform can be used to effectively amplify the *c-Myc* oncogene sequence at 0.5 ng/µL in 1.54 µL. Additionally, other isothermal amplification techniques have been integrated with DMF, presenting reaction sensitivities comparable to the one described here [6,11].

## 4. Conclusions

Successful LAMP amplification of the *c-Myc* gene fragment was achieved in the specially designed integrated DMF chip under 45 min in a total reaction volume of 1.54 µL. To the best of our knowledge, this is the first time LAMP reactions have been coupled with DMF. The developed chip enables all the typical microfluidic operations (dispensing, splitting, merging, and mixing) of the LAMP reagents and DNA solution for operating conditions of 40 V_RMS_, at 5 kHz. Droplet motion was found to be possible even for extremely low operating voltages (8 V_RMS_). Maximum and minimum droplet velocities were also determined, ranging from (2.1 ± 0.3) mm/s for 40 V_RMS_ and (0.011 ± 0.002) mm/s for 8 V_RMS_, with the present electrode configuration. This platform also houses a temperature control system integrated with a PID controller, which allows temperature stability with a maximum variance of 0.5 °C variance within the chip enclosure, thus adequate for the on-chip LAMP reactions. Additionally, the entire device can be produced by standard thin film production and patterning technologies, further lowering cost and providing large-scale integration capability including the heating element. The software and hardware controller, together with the integrated top plate inlets, makes this technology closer to an actual usable disposable platform for the real diagnostics setting since it allows for the fabrication of ready-to-use, fully automated DMF chips for nucleic acid amplification. Moreover, this technology is easily scalable with small modifications to the system controller, allowing for future multiplex target detection capabilities. Finally, an effective nucleic acid amplification platform for POC testing, would require adding extraction and purification protocols to the device. In fact, considering that the latter have been achieved on DMF, this ought to be achievable without extensive changes to the layout herein presented [30].

## Supplementary Materials

The following are available online at http://www.mdpi.com/1424-8220/17/11/2616/s1. Figure S1: Digital microfluidics device layout. (**a**) Various regions that comprise the chip, with emphasis on the zig-zag electrodes; (**b**) Photograph of the DMF chip with both bottom and top plates spaced by Kapton^®^ tape. Figure S2: DMF platform integration. An AC signal is amplified 200 times by a high voltage amplifier, and is further processed by a high voltage switching unit, which will enable either an ON or OFF state on each electrode/reservoir of the DMF platform via commands transmitted by an Arduino control board. Finally, a temperature control system assures that the on-chip temperature corresponds to the set point temperature. Figure S3: Sequential video frames, evidencing the sample input process. Figure S4: Electrophoretic analysis of the LAMP products for reaction detection capabilities, with 60 min (Lanes 2 to 7) and 90 min (Lanes 8 to 13) reaction time. Lane 1: ladder; Lane 2: 500 pg/µL initial DNA concentration; Lane 3: 50 pg/µL initial DNA concentration; Lane 4: 5 pg/µL initial DNA concentration; Lane 5: 0.5 pg/µL initial DNA concentration; Lane 6: 0.05 pg/µL initial DNA concentration; Lane 7: negative control. Lanes 8 to 13 contain the amplification products for the same order of concentration, with 90 min LAMP reaction time. Figure S5: Electrophoretic analysis of LAMP products obtained for the study of reaction volume reduction, with 60 min (Lanes 1 to 9) or 90 min (Lanes 10 to 17) reactions. The reaction volumes used in this study are as follows: 20 µL (2, 10), 15 µL (3, 11), 10 µL (4, 12), 5 µL (5, 13), 2.5 µL (6, 14), 2 µL (7, 15) and 1.25 µL (8, 16). Lane 1 corresponds to the ladder and Lanes 9 and 17 correspond to the negative controls for each reaction time (60 min and 90 min, respectively). Table S1: DMF chip electrode array layout and specifications. Table S2: Sequences of primers used for the PCR and LAMP reactions. Table S3: Material parameters for determination of heat across a DMF device. Table S4: Temperature gradient across a DMF device. Bottom plate temperatures of 60 °C and 65 °C.
