# Supplementary material for: A Digital Microfluidics Platform for Loop-Mediated Isothermal Amplification Detection
Source: Sensors (Basel). 2017 Nov 16;17(11):2616. doi: 10.3390/s17112616 (PMC5713054; doi:10.3390/s17112616)
Supplement: Supplementary file 1 [file sensors-17-02616-s001.pdf]

Supplementary Material

A Digital Microfluidics Platform for Loop-Mediated Isothermal Amplification Detection

Beatriz Jorge Coelho [1,2,†], Bruno Veigas [1,2,†], Hugo Águas [1],Elvira Fortunato [1], Rodrigo Martins [1], Pedro Viana Baptista [2,*] and Rui Igreja [1,*]

1 CENIMAT|i3N, Departamento de Ciência dos Materiais, Faculdade de Ciências e Tecnologia, Universidade NOVA de Lisboa, Campus de Caparica, 2829-516 Caparica, Portugal; bj.coelho@campus.fct.unl.pt (B.J.C.); bmrveigas@gmail.com (B.V.); hma@fct.unl.pt (H.A.); emf@fct.unl.pt (E.F.); rfpm@fct.unl.pt (R.M.)

2 UCIBIO, Departamento de Ciências da Vida, Faculdade de Ciências e Tecnologia, Universidade NOVA de Lisboa, Campus de Caparica, 2829-516 Caparica, Portugal

* Correspondence: pmvb@fct.unl.pt (P.V.B.); rni@fct.unl.pt (R.I.)

† These authors contributed equally to this work.

Received: 4 October 2017; Accepted: 10 November 2017; Published: date

Table S1. DMF chip electrode array layout and specifications[†].

Parameter	Specifications
Electrode shape	Half/full zig-zag
Electrode area (mm^2)	0.894/0.794
Electrode droplet volumes (μL)	0.2/0.1
Reservoir net area (mm^2)	9
Reservoir net volume (μL)	1.6
Gap between electrodes	30 μm
Space between bottom and top plates	180 μm

[†] The electrode specifications refer to the two electrode shapes present: 1) the half zig-zag shape, which corresponds to the electrodes nearest to the reservoirs; and 2) the full zig-zag shape, which corresponds to the remaining electrodes.

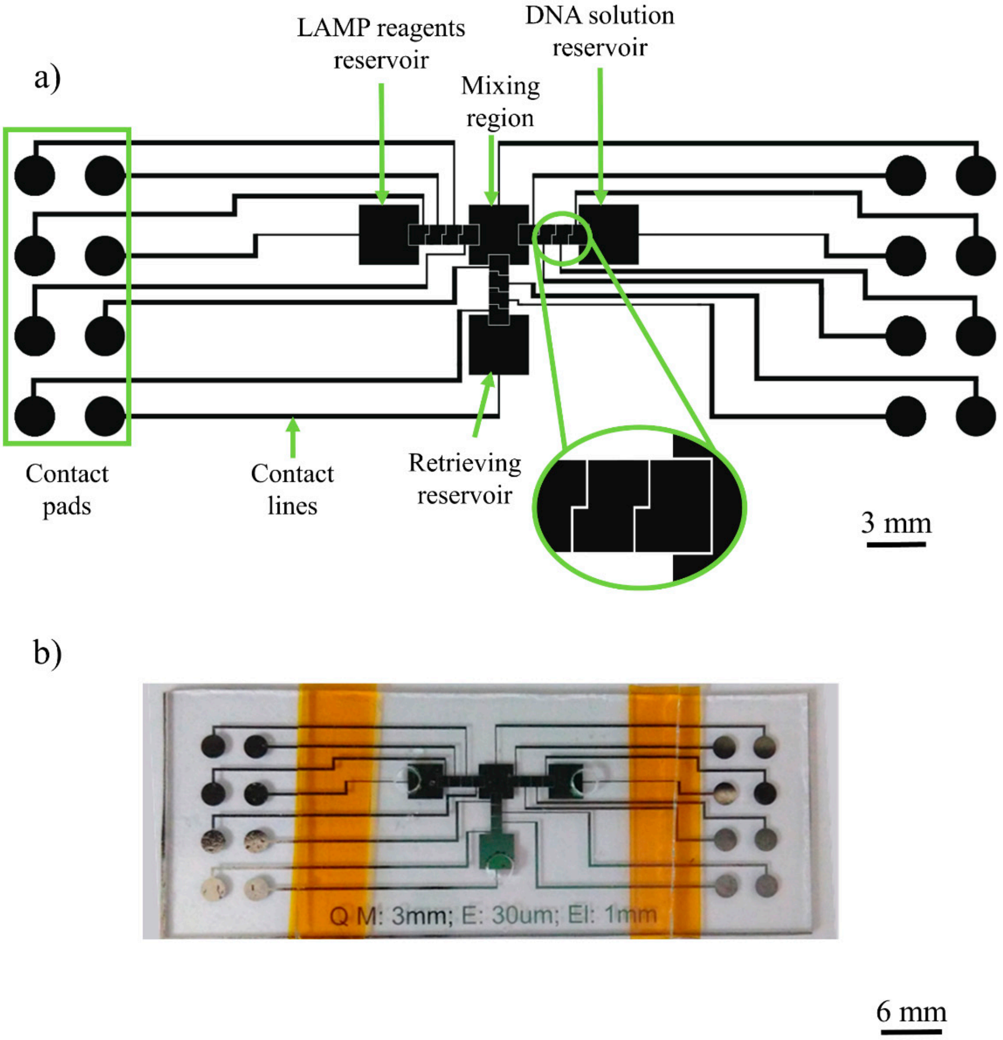

Figure S1. Digital microfluidics device layout. (a) Various regions that comprise the chip, with emphasis on the zig-zag electrodes. (b) Photograph of the DMF chip with both bottom and top plates spaced by Kapton® tape.

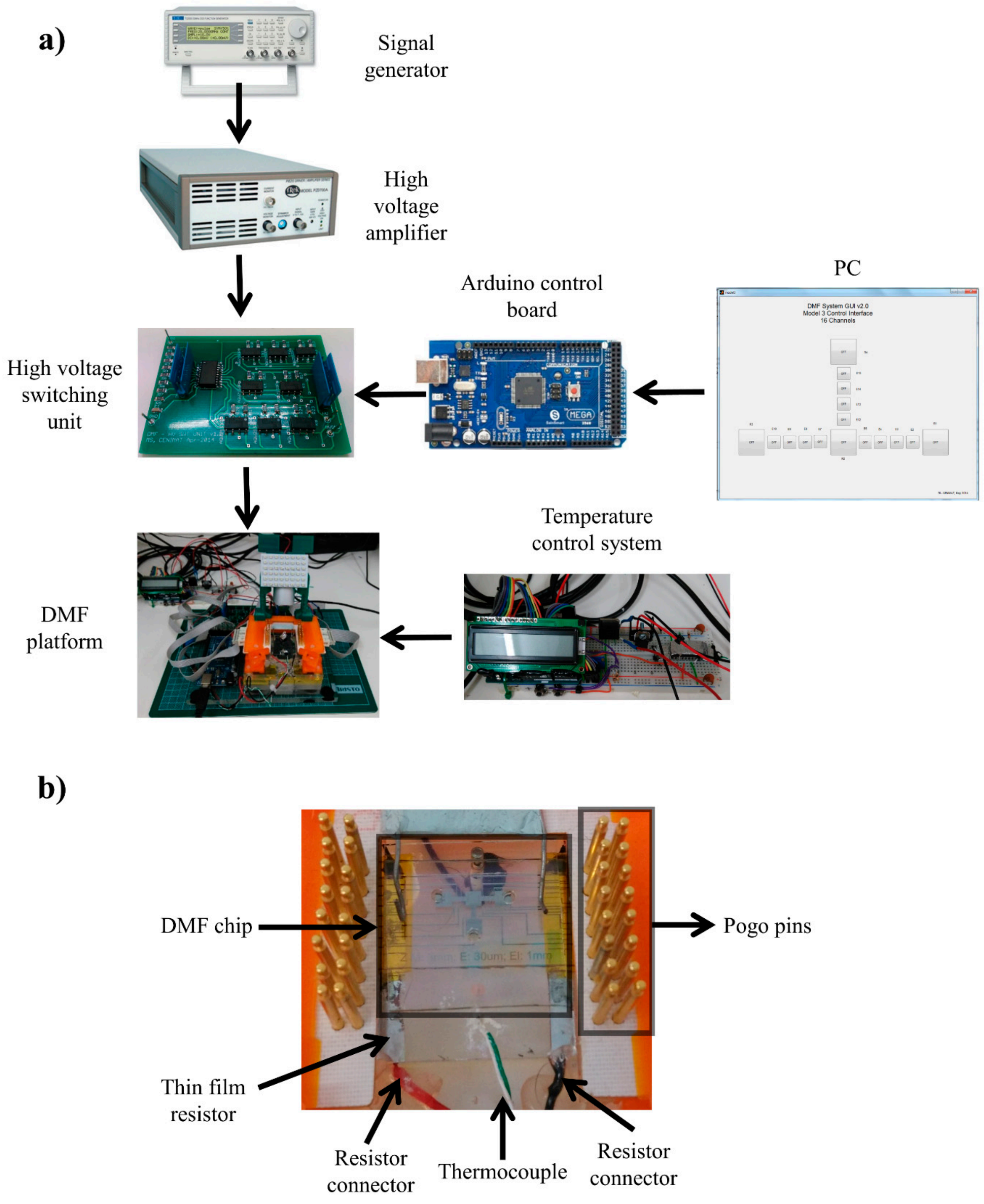

Figure S2. DMF platform integration. **a**) Overview of the DMF system. An AC signal is amplified 200 times by a high voltage amplifier, and is further processed by a high voltage switching unit, which will enable either an ON or OFF state on each electrode/reservoir of the DMF platform via commands transmitted by an Arduino control board. Finally, a temperature control system assures that the on-chip temperature corresponds to the set point temperature. **b**) Zoom on the region where the DMF chip is placed.

Table S2. Sequences of primers used for the PCR and LAMP reactions.

Primer	Sequence (5´- 3´)	Tm (°C)
	PCR Reaction	
MYCforward	GCTCATTTCTGAAGAGGAC TTGT	53.5
MYCreverse	AGGCAGTTTACATTATGGCTAAATC	52.8
	LAMP Reaction	
FP	TCTGAAGAGGACTTGTTGC	48.9
BP	TTCAGTCTCAAGACTCAGC	48.9
FIP	CTTTTCCTTACGCACAAGAGTTCC-GGAAACGACGAGAACAGT	48*
BIP	ACGATTCCTTCTAACAGAAATGTCC-CAAGGTTGTGAGGTTGCA	48*

* Melting temperature of the sequences F2 and B2 (complementary to the target sample), Notomi et al, 2000. All primers were synthesized by STAB-Vida, Portugal.

Improved sample input/output method

The improved DMF droplet input/output method was firstly tested, as well as all the fluidic operations. A top plate inlet system was developed for direct input and output of reaction reagents and products, through ports drilled on the top plate, partially overlapping the reservoirs. Figure S3 shows the droplet input process. This test was performed using 1× Bst enzyme buffer with blue dye for easier readout analysis. After sample insertion, the respective inlet reservoir is activated, allowing the droplet to spread.

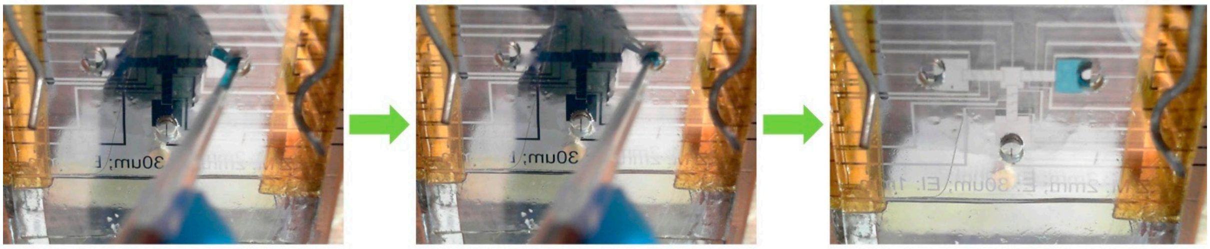

Figure S3. Sequential video frames, evidencing the sample input process.

Device working conditions and droplet speed

Regarding operating conditions, namely working voltage and frequency, this configuration allows the movement of 1× enzyme buffer droplets with 5 kHz and 40 VRMS (standard operating parameters) at reasonable speed, with the possibility of lowering voltages to 8 VRMS (see Figure 3A in the manuscripts). The movement of a solution containing DNA (0.5 ng/μL in LAMP reaction buffer) was also tested and the solution droplets were easily moved, as well as droplets containing all LAMP reagents. Droplet velocity was determined by averaging both head and tail velocities. Velocity measurements were performed by determining the amount of time the droplet took to move from one non-activated electrode to an adjacent activated electrode, considering a total motion distance of 0.83 mm.

Dynamic characterization of the temperature control system

From Figure 3B in the manuscripts, used to characterize the temperature control system, as well as the results obtained for temperature measurements at bottom (T1) and top (T5) plates, T3 was determined simply as the average between inner bottom and top plate temperatures (T2 and T4), considering that the system is approximately symmetric, which is deemed a good approximation. Furthermore, the hydrophobic, dielectric and electrode layers were omitted from all calculations and representations, since they are too thin in comparison to the glass substrates, or even the oil, and may be neglected.

The temperature rise in the thin film resistor is quite fast (200 seconds for initial temperature increase to max temperature) and heat is quickly transferred to the top plate. As temperature in the bottom plate approaches the set point temperature, there is a small overshoot, consequence of high speed controller settings, which is not visible in the top plate due to the thermal resistance of the materials between the points where T1 and T5 were measured. The PID controller is also functional, since there are no deviations from the set point temperature after reaching the stationary state.

Heat transfer for a single reaction droplet

In addition to the chip heat transfer analysis and temperature controller optimization, the temperature gradient across a LAMP reaction droplet was theoretically studied. Since on-chip LAMP reactions should occur within the optimal temperature range (60 °C to 65 °C), the droplet thermal gradient was

studied for bottom plate temperatures corresponding to both lower and higher temperature limits. Considering first a bottom plate (T1) temperature of 60 °C, to which corresponds a top plate (T5) temperature of 59.1 °C (see Figure S5), it is possible to determine the heat between both locations from an adaptation of Fourier's law, equation eqS1:

$$\frac{q}{A} = \frac{T_1 - T_5}{\frac{\Delta x_{\text{glass}}}{k_{\text{glass}}} + \frac{\Delta x_{\text{oil}}}{k_{\text{oil}}} + \frac{\Delta x_{\text{glass}}}{k_{\text{glass}}}} \qquad \text{(eqS1)}$$

Where q is the heat, Δx represents the thickness of each layer, A is the overlapping area of all layers involved and k represents the thermal conductivity for each material. Table S3 shows the various parameters of the material layers involved.

Table S3. Material parameters for determination of heat across a DMF device.

	Glass	Oil
Thickness	(0.959 ± 0.001) mm	(0.177 ± 0.001) mm
Thermal conductivity (Shackelford et al., 2016)	1.05 W/(m·K)	0.10 W/(m·K)

Solving equation eqS1, we obtain a total heat transfer per unit area (q/A) of 250.2 W/m2. Knowing the value of the heat flow, it is possible to determine any temperature from this system (T1 to T5). By determining T2 and T4, it is possible to know the gradient of temperature on a droplet during a LAMP reaction, for a bottom plate temperature of 60 °C (Table S4, set point 1). For a bottom-plate temperature of 65 °C (Table S4, set point 2), a total heat transfer per unit area of 166.9 W/m2 was determined.

Table S4. Temperature gradient across a DMF device. Bottom plate temperatures of 60 °C and 65 °C.

L	Temperature (°C)		Measurement uncertainty*
	Set point 1	Set point 2	
T1	60.0	65.0	± 0.1 °C
T2	59.8	64.9	
T3	59.6	64.7	
T4	59.3	64.6	
T5	59.1	64.4	

* Uncertainty due to the dispersion of values plus the resolution of the measuring instrument.

Reaction limit of detection

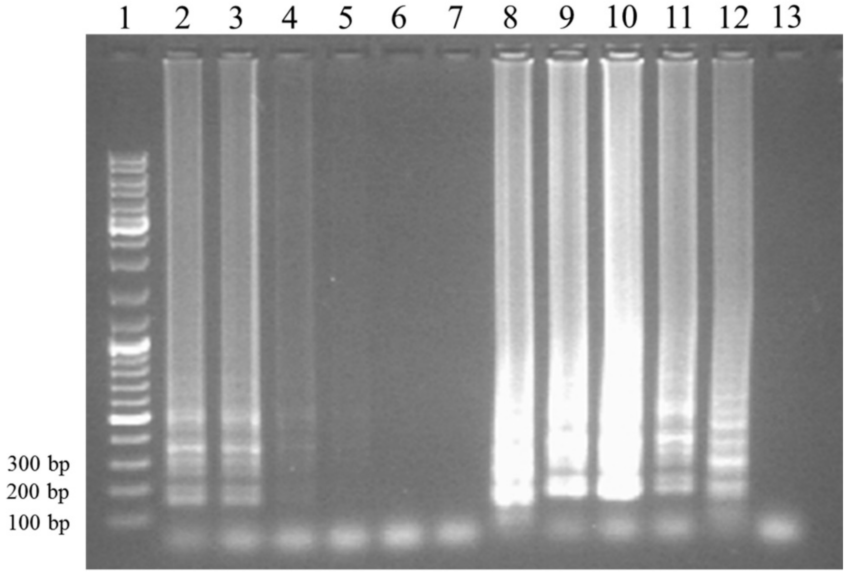

Figure S4. Electrophoretic analysis of the LAMP products for reaction detection capabilities, with 60 min. (lanes 2 to 7) and 90 min. (lanes 8 to 13) reaction time. Lane 1: ladder; lane 2: 500 pg/μL initial DNA concentration; lane 3: 50 pg/μL initial DNA concentration; lane 4: 5 pg/μL initial DNA concentration; lane 5: 0.5 pg/μL initial DNA concentration; lane 6: 0.05 pg/μL initial DNA concentration; lane 7: negative control. Lanes 8 to 13 contain the amplification products for the same order of concentration, with 90 min. LAMP reaction time.

Reaction volume

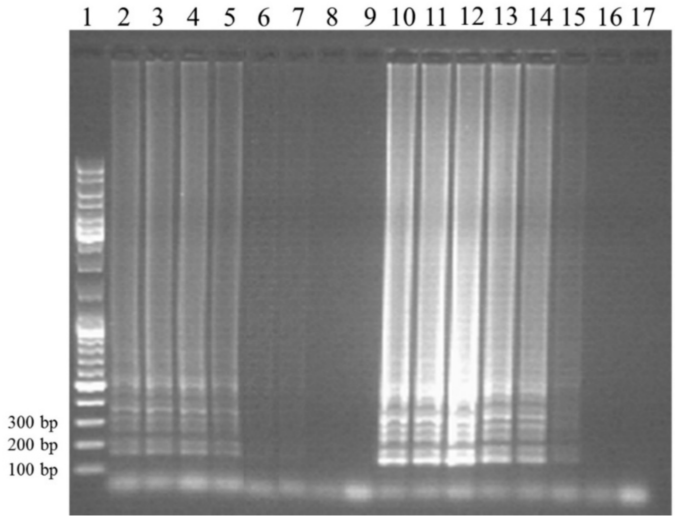

Figure S5. Electrophoretic analysis of LAMP products obtained for the study of reaction volume reduction, with 60 min. (lanes 1 to 9) or 90 min. (lanes 10 to 17) reactions. The reaction volumes used in this study are as follows: 20 μL (2, 10), 15 μL (3, 11), 10 μL (4, 12), 5 μL (5, 13), 2.5 μL (6, 14), 2 μL (7, 15) and 1.3 μL (8, 16). Lane 1 corresponds to the ladder and lanes 9 and 17 correspond to the negative controls for each reaction time (60 min. and 90 min., respectively).
